# Lipid enemas for meconium evacuation in preterm infants – a retrospective cohort study

**DOI:** 10.1186/s12887-021-02905-8

**Published:** 2021-10-18

**Authors:** Maximilian Gross, Christian F. Poets

**Affiliations:** Department of Neonatology, University Children’s Hospital Tübingen, Calwerstr. 7, 72076 Tübingen, Germany

**Keywords:** Lipid enema, Meconium, Preterm infant, Focal intestinal perforation, Necrotizing enterocolitis

## Abstract

**Background:**

Enemas are used in preterm infants to promote meconium evacuation, but frequent high-volume enemas might contribute to focal intestinal perforation (FIP). To replace a regime consisting of frequent enemas of varying volume and composition, we implemented a once-daily, low-volume lipid enema (LE) regimen. We investigated its impact on meconium evacuation, enteral nutrition, and gastrointestinal complications in preterm infants.

**Methods:**

We performed a single-center retrospective study comparing cohorts of preterm infants < 28 weeks gestation or < 32 weeks, but with birth weight < 10th percentile, before and after implementing LE. Outcomes were rates of FIP, necrotizing enterocolitis (NEC), and sepsis. We assessed stooling patterns, early enteral and parenteral nutrition. We used descriptive statistics for group comparisons and logistic regression to identify associations between LE and gastrointestinal complications and to adjust for group imbalances and potential confounders. Exclusion criteria were gastrointestinal malformations or pre-determined palliative care.

**Results:**

Data from 399 infants were analyzed, 203 before vs. 190 after implementing LE; in the latter period, 55 protocol deviations occurred where infants received no enema, resulting in 3 groups with either variable enemas, LE or no enema use. Rates of FIP and sepsis were 11.9% vs. 6.4% vs. 0.0% and 18.4% vs. 13.5% vs. 14.0%, respectively. NEC rates were 3.0% vs. 7.8% vs. 3.5%. Adjusted for confounders, LE had no effect on FIP risk (aOR 1.1; 95%CI 0.5–2.8; *p* = 0.80), but was associated with an increased risk of NEC (aOR 2.9; 95%CI 1.0–8.6; *p* = 0.048). While fewer enemas were applied in the LE group resulting in a prolonged meconium passage, no changes in early enteral and parenteral nutrition were observed. We identified indomethacin administration and formula feeding as additional risk factors for FIP and NEC, respectively (aOR 3.5; 95%CI 1.5–8.3; *p* < 0.01 and aOR 3.4; 95%CI 1.2–9.3; *p* = 0.02).

**Conclusion:**

Implementing LE had no clinically significant impact on meconium evacuation, early enteral or parenteral nutrition. FIP and sepsis rates remained unaffected. Other changes in clinical practice, like a reduced use of indomethacin, possibly affected FIP rates in our cohorts. The association between LE and NEC found here argues against further adoption of this practice.

**Trial registration:**

Registered at the German Register of Clinical Trials (no. DRKS00024021; Feb 022021).

## Introduction

Meconium passage is delayed and prolonged in preterm infants, often taking several days to complete meconium evacuation due to immaturity of the gastrointestinal tract with reduced motility and a more viscous meconium composition [1]. Neonatal care focuses on meconium evacuation because of evidence that early meconium evacuation shortens the time to full enteral feeding [2–4], subsequently reducing central venous line use and hospital stay [3, 5].

Among other interventions, enemas of varying composition have been studied in promoting meconium evacuation in preterm infants [3, 4, 6–12]. Despite limited evidence for the benefit of routinely administering enemas [13–15], they had been used in our neonatal intensive care unit (NICU) with varying frequency and volume. Given a 9.4% rate of focal intestinal perforations (FIP) in infants < 28 weeks gestational age (GA) in our NICU [16] and based on the hypothesis that frequent (high-volume) enemas contribute to FIP pathogenesis, we adopted an enema regimen in 2017 as a quality improvement initiative based on reports from another NICU with a much lower FIP rate [17].

To investigate the effects of this policy on meconium evacuation, enteral nutrition and gastrointestinal complications in our NICU, we conducted this retrospective audit.

## Materials and methods

### Study design

This analysis of historical cohorts was carried out at the NICU of Tübingen University Children’s Hospital, a tertiary perinatal center, between July 2013 and October 2020. The lipid emulsion enema protocol was implemented in April 2017. Digital patient records were analyzed. The study was registered at the German Register of Clinical Trials (trial no. DRKS 00024021) and was approved by the ethics committee of Tübingen University Hospital (application no. 965/2020BO), including an informed consent waiver.

### Patients

Analysis included all preterm infants < 28 weeks GA or < 32 weeks GA with a birth weight < 10th percentile admitted to our NICU within the first 24 postnatal hours. Infants with gastrointestinal malformations or pre-determined palliative care were excluded. We used growth charts based on national perinatal data for somatic classification of newborns [18].

### Routine care and interventions to promote meconium evacuation

Respiratory care was performed according to international standards [19, 20]. We used two drug regimens for the prevention of bronchopulmonary dysplasia (BPD): (i) systemic hydrocortisone (cumulative dose 8.5 mg/kg) during the first ten postnatal days in infants < 28 weeks GA and birth weight > 3rd percentile [21], or (ii) inhaled budesonide (800 μg/d on days 1–14, followed by 400 μg/d until 32 weeks postmenstrual age) in infants < 28 weeks GA with birth weight < 3rd percentile receiving nasal continuous positive airway pressure or intermittent positive pressure ventilation [22]. Caffeine therapy was started on the day of birth. Isotonic fluid boluses, noradrenaline or norepinephrine and hydrocortisone were used to treat arterial hypotension, as needed. We used indomethacin and ibuprofen for pharmacologic closure of a hemodynamically significant persistent ductus arteriosus (PDA). Concurrent use of cyclooxygenase (COX) inhibitors and systemic corticosteroids was avoided by maintaining a minimum time interval of at least 12 h between both drugs. A standardized enteral feeding protocol was applied to all preterm infants using bolus feeds given at 2-h intervals, starting on day one with formula or (donated) expressed breast milk at a volume of 25 to 30 ml/kg/d with daily increments of 30 ml/kg irrespective of the type of milk. Full enteral feeds were defined as a daily intake of 150 ml/kg. Parenteral nutrition was initiated immediately after birth following European Society for Paediatric Gastroenterology, Hepatology and Nutrition (ESPGHAN) guidelines [23]. Intravenous (IV) parenteral nutrition was discontinued once enteral intake reached 130 to 150 ml/kg/d.

Before implementing lipid enemas, no standardized enema protocol existed, and enemas were administered at nurses’ and physicians’ discretion with varying substances, including normal saline, Tween80 [8], glycerin [3], and mixtures of the above. Applied volumes (10 to 40 ml/kg per enema) and frequencies (1-3x/day) varied widely.

Lipid enemas were introduced in April 2017 and meant to be applied to all preterm infants < 28 weeks GA and those < 32 weeks GA with a birth weight < 10th percentile admitted thereafter (Table 1). The adopted concept included use of an intravenous lipid emulsion as enema solution and standardization of its administration, based on the following pathophysiologic considerations: (i) use of a sterile solution to minimize bacterial contamination, (ii) oil to soften up the meconium, (iii) a fixed volume (10 ml/kg) and (iv) duration of administration (10 to 15 min) to avoid overdistension of the intestinal wall and achieve proper mixing of the lipid solution with meconium, (v) a plug-like application device that avoids deep rectal catheter insertions. We administered lipid enemas once every 24 h until the passage of transitional stool. Based on individual preference of the attending neonatologist, other enemas could be administered. An iodine-based non-ionic low osmolar contrast agent (Ultravist® 300, Bayer Vital GmbH, Leverkusen, Germany) was used in both cohorts for the diagnostic workup in case of absent meconium evacuation on postnatal days 3–5.

**Table 1:** In-house guideline for meconium mobilization starting in April 2017

Preterm infants < 28 weeks gestational age or < 32 weeks gestational age if birth weight is <10th percentile: • Apply the first enema not earlier than 24 h after birth • Use special rectal catheter (Prirektosilk natal®, Primed, Halberstadt, Germany) • Insert catheter for no more than 3 cm • Instill warmed lipid emulsion (SMOFlipid®20%, Fresenius Kabi AG, Bad Homburg, Germany) using a syringe • Apply a volume of 10 ml/kg • Apply lipid emulsion slowly over 10 to 15 min. Leave catheter in place for a few minutes • Remove catheter • If necessary, apply gentle abdominal massage afterward • Repeat once daily until the infant passes transitional stool	

### Study objectives

The primary outcome was the FIP rate before and after implementing the above protocol. Secondary outcomes were (i) days from birth to full enteral feeds (enteral intake ≥150 ml/kg/d), (ii) days with IV access for parenteral nutrition, (iii) number of enemas administered and (iv) of stools until the passing of normal stool, (v) number of infants with necrotizing enterocolitis ≥ Bell stage IIA, (vi) number of infants with a diagnosis of sepsis according to the criteria used by the national surveillance system for infections in very low birth weight infants [24].

### Statistical analysis

To detect a clinically relevant 5% decrease in FIP rates of from 9.4% (effect size ω = 0.18; alpha 0.05; power 0.80), we calculated an overall sample size of 401 patients using G*Power Version 3.1.9.7 [25]. We reported the basic patient data and influencing factors for FIP based on the date of implementing lipid enemas (before vs. after) and actual treatment (variable enemas vs. lipid enemas vs. no enemas). Group differences were evaluated using t-test for normally distributed and Mann-Whitney-U-Test for non-normally distributed data, and chi-square test or Fisher’s exact test for categorical items. Statistical significance was set at *p* < 0.05. Statistical analysis was done using logistic regression (outcome: occurrence of FIP or NEC) and adjusting for extremely low birth weight status (< 1000 g), low gestational age (< 28 weeks), administration of lipid enemas, and group imbalances (each coded as yes or no depending on present or absent). Possible collinearity and interactions with influencing factors were tested. No adjustment for multiple testing was made. All *p*-values are descriptive. Analyses were performed using Statistical Package for Social Sciences Version 25 (IBM Corp., Armonk, NY, USA).

## Results

We screened 452 infants < 28 weeks GA or < 32 weeks GA with birth weight < 10th percentile for inclusion in this analysis; 53 were excluded (40 outborns; 9 receiving palliative care only; 3 with gastrointestinal malformations; 1 with transfer to another NICU on postnatal day 3). Three hundred ninety nine infants were available for further analysis, 203 before and 196 after implementing lipid enemas (Table 2).

**Table 2:** Patients’ characteristics before vs. after implementing lipid enemas (“intention to treat analysis”)

	Before implementing lipid enemas (*n* = 203)	After implementing lipid enemas (*n* = 196)	*P*-value
Gestational age; mean (weeks)	27.1 (2.1)	27.0 (2.2)	0.67
Gestational age; median (weeks)	26.9 (23.3–31.9)	26.9 (23.4–31.9)	
Birth weight; mean (g)	764.5 (214.4)	782.3 (210.0)	0.41
Birth weight; median (g)	745.0 (280–1340)	772.5 (240–1240)	
Male	103/203 (50.7%)	98/196 (50.0%)	0.88
Small for gestational age	84/203 (41.4%)	79/196 (40.3%)	0.83
Multiple births	86/203 (42.4%)	67/196 (34.2%)	0.09
Caesarean section	187/203 (92.1%)	181/196 (92.3%)	0.93
Antenatal corticosteroid therapy	190/203 (93.6%)	175/196 (89.3%)	0.12
Pre-eclampsia	13/203 (6.4%)	18/196 (9.2%)	0.30
Oligohydramnios	16/203 (7.9%)	6/196 (3.1%)	**0.04**
Chorioamnionitis	48/203 (23.6%)	37/196 (18.9%)	0.25
Apgar 1	5 (0–9)	6 (0–9)	**0.04**
Apgar 5	8 (0–10)	8 (2–10)	0.84
Apgar 10	9 (1–10)	9 (4–10)	0.23
pH at birth	7.3 (6.83–7.49)	7.3 (6.85–7.48)	0.85
No. of surfactant doses	1 (0–6)	1 (0–5)	0.45
Moderate or severe BPD	32/203 (15.8%)	29/196 (14.8%)	0.79
Treatment with dexamethasone for BPD	16/203 (7.9%)	13/196 (6.6%)	0.63
Treatment with hydrocortisone:			
for arterial hypotension	30/203 (14.8%)	25/196 (12.8%)	0.56
for BPD prophylaxis	1/203 (0.5%)	99/196 (50.5%)	**< 0.01**
Treatment w. inhaled budesonide during first 14 days	66/203 (32.5%)	39/196 (19.9%)	**< 0.01**
PDA treatment with Indomethacin	90/203 (44.3%)	27/196 (13.8%)	**< 0.01**
PDA treatment with Ibuprofen	44/203 (21.7%)	46/196 (23.5%)	0.67
PDA ligation	5/203 (2.5%)	2/196 (1.0%)	0.27
IVH grades 1 to 3	32/203 (15.8%)	25/196 (12.7%)	0.39
Periventricular hemorrhagic infarction	9/203 (4.4%)	11/196 (5.6%)	0.59
PVL	5/203 (2.5%)	2/196 (1.0%)	0.27
ROP treatment (laser/antibody)	4/203 (2.0%)	4/196 (2.0%)	0.96
Fed predominantly with EBM during first 14 days	107/203 (52.7%)	134/196 (68.4%)	**< 0.01**
Duration of hospital stay (days)	74 (1–232)	72 (1–232)	0.69
Weight at discharge (g)	2523.4 (887.7)	2566.5 (819.7)	0.58
Mortality	20/203 (9.9%)	17/196 (8.7%)	0.69

Data presented as mean (± standard deviation); median (minimum and maximum), or as the number of patients with percentages in parenthesis. *BPD* Bronchopulmonary dysplasia, *DOL* Day of life, *EBM* Expressed breast milk, *IVH* Intraventricular hemorrhage, *PDA* Patent ductus arteriosus, *PVL* Periventricular leukomalacia, *ROP* Retinopathy of prematurity

In the cohort preceding lipid enema implementation, 201 infants received between two and 41 enemas (median 10.0) until the passing of normal stool, and two infants received none. Enemas consisted of normal saline, Tween80, glycerin, and mixtures of the above. Protocol adherence, i.e., actual treatment with lipid enemas after implementing this policy, was 71.9% (141 of 196 eligible infants). Of these 141 infants, 42 (30%) received between one and four (median 1.0) additional saline enemas, accounting for 9.9% of the total number of enemas in this group. After implementing lipid enemas, 55 infants received no enemas, i.e., had to be regarded as protocol deviations and were thus analyzed separately. Patient data and influencing factors for FIP stratified by treatment with variable enemas vs. lipid enemas vs. no enemas are presented in Table 3.

**Table 3:** Patients’ characteristics stratified by actual treatment applied (variable enemas, lipid enemas, no enemas)

	(I) Variable enemas (*n* = 201)	(II) Lipid enemas (*n* = 141)	(III) No enemas (*n* = 57)	*P*-values
Gestational age; mean (weeks)	27.1 (2.2)	26.9 (2.1)	27.3 (2.3)	I vs. II 0.45 I vs. III 0.57 II vs. III 0.28
Gestational age; median (weeks)	26.9 (23.3–31.7)	26.7 (23.4–31.9)	26.9 (23.6–31.9)	
Birth weight; mean (g)	760.5 (211.6)	764.3 (214.4)	840.4 (199.5)	I vs. II 0.85 I vs. III 0.20 II vs. III **0.04**
Birth weight; median (g)	745 (280–1060)	740 (240–1230)	795 (490–1240)	
Male	99/201 (49.3%)	75/141 (53.2%)	24/57 (42.1%)	I vs. II 0.47 I vs. III 0.34 II vs. III 0.16
Small for gestational age	84/201 (41.8%)	57/141 (40.4%)	22/57 (38.6%)	I vs. II 0.80 I vs. III 0.67 II vs. III 0.81
Multiple births	86/201 (42.8%)	47/141 (33.4%)		I vs. II 0.08 I vs. III 0.29 II vs. III 0.81
Caesarean section	185/201 (92.0%)	131/141 (92.2%)	52/57 (91.1%)	I vs. II 0.77 I vs. III 0.84 II vs. III 0.69
Antenatal corticosteroid therapy	188/201 (93.5%)	127/141 (90.1%)	50/57 (87.8%)	I vs. II 0.24 I vs. III 0.15 II vs. III 0.63
Pre-eclampsia	13/201 (6.5%)	13/141 (9,2%)	5/57 (8.8%)	I vs. II 0.35 I vs. III 0.55 II vs. III 0.92
Oligohydramnios	15/201 (7.5%)	5/141 (3.5%)	2/57 (3.5%)	I vs. II 0.13 I vs. III 0.29 II vs. III 0.99
Chorioamnionitis	47/201 (23.4%)	25/141 (17.7%)	13/57 (22.8%)	I vs. II 0.21 I vs. III 0.93 II vs. III 0.41
Apgar 1	5 (1–9)	6 (0–9)	6 (0–9)	I vs. II 0.17 I vs. III 0.11 II vs. III0.63
Apgar 5	8 (1–10)	8 (2–10)	8 (0–10)	I vs. II 0.28 I vs. III 0.18 II vs. III **0.04**
Apgar 10	9 (1–10)	9 (4–10)	9 (1–10)	I vs. II 0.92 I vs. III **0.03** II vs. III **0.04**
pH at birth	7.3 (6.83–7.49)	7.3 (6.85–7.48)	7.3 (7.02–7.40)	I vs. II 0.58 I vs. III 0.66 II vs. III 0.40
No. of surfactant doses	1.0 (0–6)	1 (0–5)	1 (0–3)	I vs. II 0.73 I vs. III 0.61 II vs. III **0.04**
Moderate or severe BPD	32/201 (15.9%)	23/141 (16.3%)	6/57 (10.5%)	I vs. II 0.92 I vs. III 0.31 II vs. III 0.29
Treatment with dexamethasone for BPD	16/201 (8.0%)	10/141 (7.1%)	3/57 (5.3%)	I vs. II 0.77 I vs. III 0.49 II vs. III 0.64
Hydrocortisone for arterial hypotension	30/201 (14.9%)	20/141 (14.2%)	5/57 (8.8%)	I vs. III 0.85 I vs. III 0.23 II vs. III 0.29
Hydrocortisone for BPD prophylaxis	1/201 (0.5%)	70/141 (49.6%)	29/57 (50.9%)	I vs. II < 0.01 I vs. III < 0.01 II vs. III < 0.89
Treatment w. inhaled budesonide during first 14 days	66/201 (32.8%)	29/141 (20.6%)	10/57 (17.5%)	I vs. II **0.01** I vs. III **0.03** II vs. III 0.63
PDA treatment with Indomethacin	90/201 (44.8%)	23/141 (16.3%)	4/57 (7.0%)	I vs. II **< 0.01** I vs. III **< 0.01** II vs. III 0.08
PDA treatment with Ibuprofen	44/201 (21.9%)	35/141 (24.8%)	11/57 (19.3%)	I vs. II 0.53 I vs. III 0.67 II vs. III 0.41
PDA ligation	5/201 (2.5%)	2/141 (1.4%)	0/57 (0.0%)	I vs. II 0.49 I vs. III 0.23 II vs. III 0.37
IVH grades 1 to 3	32/201 (15.9%)	21/141 (14.9%)	4/57 (7.0%)	I vs. II 0.80 I vs. III 0.09 II vs. III 0.13
PVHI	9/201 (4.5%)	7/141 (5.0%)	4/57 (7.0%)	I vs. II 0.83 I vs. III 0.44 II vs. III 0.57
PVL	5/201 (2.5%)	2 (1.4%)	0/57 (0.0%)	I vs. II 0.49 I vs. III 0.23 II vs. III 0.37
ROP treatment (laser/antibody)	4/201 (2.0%)	3/141 (2.1%)	1/57 (1.8%)	I vs. II 0.93 I vs. III 0.91 II vs. III 0.87
Fed predominantly with EBM during first 14 days	105/201 (52.2%)	100/141 (70.9%)	35/57 (61.4%)	I vs. II **< 0.01** I vs. III 0.22 II vs. III 0.19
Duration of hospital stay (days)	74.0 (1–232)	73 (1–225)	65 (1–232)	I vs. II 0.81 I vs. III 0.19 II vs. III 0.26
Weight at discharge (g)	2538.9 (881.6)	2458.7 (845.6)	2457.9 (782.9)	I vs. II 0.57 I vs. III 0.37 II vs. III 0.54
Mortality	18/201 (9.0%)	13/141 (9.2%)	6/57 (10.5%)	I vs. II 0.93 I vs. III 0.72 II vs. III 0.78

Data presented as mean (± standard deviation); median (minimum and maximum), or as the number of patients with percentages in parenthesis. *BPD* Bronchopulmonary dysplasia, *DOL* Day of life, *EBM* Expressed breast milk, *IVH* Intraventricular hemorrhage, *PDA* Patent ductus arteriosus, *PVHI* Periventricular hemorrhagic infarction, *PVL* Periventricular leukomalacia, *ROP* Retinopathy of prematurity

Contrast agent was administered enterally to a total of 43 infants (13.3% before vs. 8.2% after implementing lipid enemas; *p* = 0.10). Treatment with indomethacin and inhaled budesonide was reported more often in the cohort before implementing lipid enemas, whereas treatment with hydrocortisone for BPD prophylaxis and expressed breast milk feeding were more common in the cohort afterward. Infants without enema treatment tended to have a higher birth weight.

Reported FIP rates were not significantly lower in infants receiving lipid enemas (Table 4), and the latter were not associated with a lower risk of FIP in uni- or multivariable analysis (odds ratio (OR) 0.67; 95% confidence interval (CI) 0.3–1.5; *p* = 0.31; adjusted (a) aOR 1.1; 95%CI 0.5–2.8; *p* = 0.80). We adjusted for GA, birth weight, indomethacin, budesonide and hydrocortisone administration as well as expressed breast milk feeding. Indomethacin treatment, however, was associated with an increased risk of FIP in our cohorts after adjustment for the above potential confounders (aOR 3.5; 95%CI 1.5–8.3; *p* < 0.01).

**Table 4:** Primary and secondary outcomes of infants treated with variable enemas, lipid enemas and no enemas

	(I) Variable enemas (*n* = 201)	(II) Lipid enemas (*n* = 141)	(III) No enemas (*n* = 57)	*P*-values
**Focal intestinal perforation**				
FIP rate	24/201 (11.9%)	9/141 (6.4%)	0/57(0.0%)	I vs. II 0.09 I vs. III **< 0.01** II vs. III 0.06
Diagnosis of FIP (DOL)	6 (2–20)	12 (5–21)	–	I vs. II 0.23 I vs. III - II vs. III -
Location of FIP				
- Terminal ileum	19/24 (79.2%)	5/9 (55.6%)	–	I vs. II **0.04**
- Colon	3/24 (12.5%)	4/9 (44.4%)	–	I vs. III 0.39
- Not specified or no surgical treatment	2/24 (8.3%)	0	–	
**Necrotizing enterocolitis and sepsis**				
NEC rate	6/201 (3.0%)	11/141 (7.8%)	2/57 (3.5%)	I vs. II **0.04** I vs. III 0.84 II vs. III 0.35
Diagnosis of NEC (DOL)	12.5 (6–46)	7 (3–44)	7 (4–10)	I vs. II 0.59 I vs. III 0.29 II vs. III 0.51
Diagnosis of ≥1 sepsis	37/201 (18.4%)	19/141 (13.5%)	8/57 (14.0%)	I vs. II 0.23 I vs. III 0.44 II vs. III 0.92
**Stooling pattern and sum of enemas**				
First meconium (DOL)	1 (1–2)	2 (1–5)	1 (1–3)	I vs. II **< 0.01** I vs. III 0.24 II vs. III **< 0.01**
First transitional stool (DOL)	5 (2–14)	5 (3–16)	5 (2–9)	I vs. II **0.04** I vs. III 0.06 II vs. III **< 0.01**
First normal stool (DOL)	7 (4–20)	7 (5–20)	6.5 (5–16)	I vs. II **< 0.01** I vs. III 0.97 II vs. III **< 0.01**
Sum of stools until first normal stool	20 (9–59)	19 (6–57)	20 (9–38)	I vs. II 0.19 I vs. III 0.64 II vs. III 0.62
Sum of enemas until first normal stool	10 (2–41)	3 (1–22)	0	I vs. II **< 0.01** I vs. III **< 0.01** II vs. III **< 0.01**
**Enteral and parenteral nutrition**				
Days from birth to full enteral feeds	7 (5–110)	7 (5–47)	6 (4–98)	I vs. II 0.05 I vs. III **< 0.01** II vs. III **< 0.01**
Enteral intake on the 14th day of life (ml)	154 (0–186)	154 (0–186)	157 (100–175)	I vs. II 0.66 I vs. III 0.34 II vs. III 0.53
Days from birth with IV line for PN	7 (5–109)	7 (5–79)	6 (5–98)	I vs. II 0.13 I vs. III **< 0.01** II vs. III **< 0.01**
N Infants with central IV lines	97/201 (48.3%)	79/141 (56.0%)	18/57 (31.6%)	I vs. II 0.16 I vs. III **0.03** II vs. III **< 0.01**
Total days with central IV lines	8 (1–109)	8 (1–163)	6.5 (1–32)	I vs. II 0.27 I vs. III **0.01** II vs. III **< 0.01**

Data presented as mean (± standard deviation); median (minimum and maximum), or as the number of patients with percentages in parenthesis. *DOL* Day of life, *FIP* Focal intestinal perforation, *NEC* Necrotizing enterocolitis, IV Intravenous, *PN* Parenteral nutrition

Concerning secondary outcomes (Table 4), time to full enteral feeds and days with parenteral nutrition did not differ between variable and lipid enema groups. However, infants not receiving any enema had a reduced time to full enteral feeds and fewer days with parenteral nutrition. Fewer enemas were administered in the lipid enemas group, and a delayed passage of meconium was observed without altering the overall number of stools. Infants not receiving any enema showed a faster meconium passage. NEC rates were higher in infants treated with lipid enemas. After adjustment for GA, birth weight, indomethacin, budesonide, hydrocortisone administration, and expressed breast milk feeding, lipid enemas were associated with an increased risk of NEC (aOR 2.9; 95%CI 1.0–8.6; *p* = 0.048). Also, formula feeding was an independent risk factor for NEC (aOR 3.4; 95%CI 1.2–9.3; *p* = 0.02).

## Discussion

This retrospective cohort study audited the effects of early postnatal lipid enemas on stooling patterns, enteral and parenteral nutrition, and gastrointestinal complications in very preterm infants.

FIP rates were not significantly decreased after implementing lipid enemas (or no enemas), and we found no association between lipid enema use and FIP risk.

The pathogenesis of FIP is still unclear [26, 27]. Several risk factors like chorioamnionitis or exposure to glucocorticoids and indomethacin have been identified [28–31]. These factors may predispose to FIP through reduced perfusion and collagen synthesis disturbances in specific regions of the intestinal wall [27]. In our cohort, exposure to glucocorticoids was higher in the lipid enema group without affecting FIP rates. However, treatment with indomethacin was associated with an increased risk of FIP, which is in line with results from other studies [29]. Indomethacin was administered less frequently in the cohort receiving lipid enemas due to a more restrictive approach to PDA treatment in our unit in recent years.

Also, infants treated with lipid enemas were more often predominantly fed expressed breast milk after implementing lipid enemas, which resulted from the introduction of donor human milk in our NICU in 2018. A high breast milk intake in the first postnatal week may be associated with a lower risk of FIP (own unpublished data), but we did not find an association between the type of feeding and FIP risk in our cohort. Sharpe et al. reported a lower rate of non-NEC associated gastrointestinal perforations in a retrospective cohort analysis after implementing probiotics and donor human milk in their NICU, but differences were not statistically significant [32].

Whether our considerations leading to the implementation of lipid enemas influenced the risk of FIP remains unproven; to our knowledge, there is no evidence linking enemas to FIP. At the same time, intestinal perforation represents a procedural complication of enema use in preterm infants [33]. Reducing either bacterial contamination or intestinal wall distension using a sterile, low-volume enema solution seemed reasonable as sepsis often accompanies FIP [28], and both, viscous meconium and an excessive fluid volume, may further weaken an already compromised intestinal wall. Our low-volume lipid enemas are unlikely to have reached the ascending colon and terminal ileum [2], the primary sites of FIP, but rather promoted meconium evacuation from the distal colon. The latter, combined with transient rectal distension, which increases small intestine transit time in adults [34], might help to improve early postnatal gastrointestinal function, but again, this is an unproven hypothesis [15]. Likewise, it is debatable whether small-volume enemas help prevent meconium obstruction, a specific clinical condition of very low birth weight infants, for which enemas are used therapeutically [35, 36].

FIP occurred predominantly in the terminal ileum in our cohorts. We may have included perforations provoked by functional intestinal obstruction of prematurity, a disease spectrum that can resemble FIP and is not easily distinguishable [37]. Also, in two cases (one in each cohort), perforation might have been iatrogenic as rapid clinical deterioration occurred shortly after enema application. Due to the retrospective nature of our analysis, we could not investigate these potential procedural complications further. All infants with FIP occurring after implementing lipid enemas underwent surgical treatment with histopathologic confirmation of the clinical diagnosis. No cases of Hirschsprung’s disease or cystic fibrosis were reported in infants diagnosed with FIP.

NEC rates increased from the first to the second period, with overall rates still in line with data from a comparable population [38]. In multivariable analysis, treatment with lipid enemas and being fed predominantly with formula during the first postnatal days were associated with an increased risk of NEC. While prioritizing feeding with expressed breast milk is an established intervention to reduce NEC [39], there are conflicting data regarding an association between enemas and NEC. In a meta-analysis from 2015, including one study using small-volume enemas (10 ml/kg normal saline mixed with glycerin), a “trend toward increased risk of NEC” was reported in infants treated with glycerin enemas and suppositories [13]. A meta-analysis from 2016 of six RCTs, including three studies using enemas (normal saline, glycerin, or both), found no increased NEC risk through enema use [15]. However, one of the included studies reported a 23% NEC rate in the intervention group receiving 10 ml/kg saline enemas and rectal stimulation twice daily, compared to 3% in controls [10]. In a more recent study, no NEC ≥ IIA Bell stage occurred in 28 infants treated with normal saline enemas during the first postnatal days [12]. How enemas potentially increase NEC risk remains unclear, including whether this potential association is due to the enemas’ composition.

In the second observation period, increased NEC rates and a lack of continuous staff training may have reduced protocol adherence. The high proportion of infants *not* receiving enemas despite being eligible only became apparent to us during analysis of this study’s data, leading to a subdivision of the cohorts to separately provide data on infants not receiving any enema treatment (before implementing LE, only two infants received no enemas as they died soon after birth). A more restrictive approach to enemas was only observed in the cohort after implementing lipid enemas and may have been a result of discussions during this practice’s implementation. We observed a faster meconium passage, shorter time with an IV line, and shorter time to full enteral feeds in infants *not* receiving any enema, despite being eligible. These infants had a higher birth weight and appeared to undergo a more rapid postnatal gastrointestinal adaptation which may have led to enemas being considered unnecessary by the unit staff, suggesting a bias by indication.

The implementation of lipid enemas led to a significant decrease in overall use of enemas and prolonged meconium passage time in infants who were treated with lipid enemas, without increasing the number of days to full enteral feeds or days with an IV line for parenteral nutrition. Central venous line use was higher in the lipid enema cohort, likely related to its higher NEC rate.

Considering our results and the low evidence for routinely administering enemas to promote meconium evacuation and enteral feeding [15], it is conceivable that many very preterm infants may not need routine assistance with meconium evacuation and may be allowed to undergo gastrointestinal adaptation on their own.

Our study has several limitations. The sample size may have been too small to detect an effect of lipid enemas on FIP rates, although, even with a larger sample size, it seems more plausible that changes in practice other than lipid enemas influenced FIP rates. Our study covered a timespan of over 7 years, in which several other changes occurred, including a reduced use of indomethacin, increased use of low-dose hydrocortisone, and the introduction of donor milk. There is the possibility of bias caused by the proportion of infants in the lipid enema group that additionally received saline enemas. Administering additional saline enemas was allowed by the in-house lipid enema guideline and can therefore not be seen as a protocol violation. Because of the small proportion of saline enemas vs. total enemas administered in the lipid enema group, we refrained from excluding these infants from the lipid enema cohort to avoid further fragmentation of our study groups. Non-compliance with the protocol was high as many infants did not receive any enema after implementing lipid enemas despite being eligible, necessitating a further breakdown of the cohorts. We did not report further potential influencing factors on FIP rate, like antenatal treatment with magnesium sulfate or the timing of antenatal glucocorticoid therapy, as these data were not consistently documented in the patient charts. With its retrospective design, it is inherent that our study did not sufficiently analyze additional factors crucial to preterm infant care. In both cohorts, enema use was usually discontinued once transitional stool occurred. Since identifying transitional stool may sometimes be difficult and rater-dependent, we chose normal stool as an endpoint for comparing the sum of stools and applied enemas.

## Conclusion

Implementing lipid enemas to promote meconium evacuation was not associated with the desired reduction in FIP rate, but led to the application of fewer enemas, affecting meconium transit time without clinical consequences. Treatment with indomethacin was associated with a higher risk of FIP. Also, we detected an association between lipid enemas and an increased risk of NEC, although all of these findings can only be hypothesis-generating. Large, randomized controlled trials are needed to evaluate the routine use of enemas in preterm infants. Without clear benefit and bearing potential for harm, we are currently reviewing the use of lipid enemas with the prospect of abandoning this practice altogether.

## Data Availability

The data presented in this study are available on request form the corresponding author.

## Abbreviations

BPD: Bronchopulmonary dysplasia
COX: Cyclooxygenase
d: Day
DOL: Day of life
EBM: Expressed breast milk
ESPGHAN: European Society for Paediatric Gastroenterology, Hepatology and Nutrition
FIP: Focal intestinal perforation
GA: Gestational age
h: Hour
IV: Intravenous
IVH: Intraventricular hemorrhage
LE: Lipid enema
NEC: Necrotizing enterocolitis
NICU: Neonatal intensive care unit
PDA: Persistent ductus arteriosus
PN: Parenteral nutrition
PVL: Periventricular leukomalacia
ROP: Retinopathy of prematurity
wk.: Week
